# Evaluation of Novel Dual Acetyl- and Butyrylcholinesterase Inhibitors as Potential Anti-Alzheimer’s Disease Agents Using Pharmacophore, 3D-QSAR and Molecular Docking Approaches

**DOI:** 10.3390/molecules22081254

**Published:** 2017-07-26

**Authors:** Xiaocong Pang, Hui Fu, Shilun Yang, Lin Wang, Ai-Lin Liu, Song Wu, Guan-Hua Du

**Affiliations:** 1 Institute of Material Medical, Chinese Academy of Medical Sciences and Peking Union Medical College, Xian Nong Tan Street, Beijing 100050, China; pangxiaocong@imm.ac.cn (X.P.); wlin@imm.ac.cn (L.W.); 2 Beijing Institute for Drug Control, Beijing 102206, China; fuhuifree@sina.com; 3 Department of Pharmacology, Shenyang Pharmaceutical University, Shenyang 110016, China; yangsl@imm.ac.cn; 4 Beijing Key Laboratory of Drug Target Research and Drug Screening, Chinese Academy of Medical Sciences and Peking Union Medical College, Beijing 100050, China; 5 State Key Laboratory of Bioactive Substance and Function of Natural Medicines, Chinese Academy of Medical Sciences and Peking Union Medical College, Beijing 100050, China

**Keywords:** cholinesterase inhibitor, Alzheimer’s disease, DL0410, 3D-QSAR, molecular docking, kinetics

## Abstract

DL0410, containing biphenyl and piperidine skeletons, was identified as an acetylcholinesterase (AChE) and butyrylcholinesterase (BuChE) inhibitor through high-throughput screening assays, and further studies affirmed its efficacy and safety for Alzheimer’s disease treatment. In our study, a series of novel DL0410 derivatives were evaluated for inhibitory activities towards AChE and BuChE. Among these derivatives, compounds **6-1** and **7-6** showed stronger AChE and BuChE inhibitory activities than DL0410. Then, pharmacophore modeling and three-dimensional quantitative structure activity relationship (3D-QSAR) models were performed. The R^2^ of AChE and BuChE 3D-QSAR models for training set were found to be 0.925 and 0.883, while that of the test set were 0.850 and 0.881, respectively. Next, molecular docking methods were utilized to explore the putative binding modes. Compounds **6-1** and **7-6** could interact with the amino acid residues in the catalytic anionic site (CAS) and peripheral anionic site (PAS) of AChE/BuChE, which was similar with DL0410. Kinetics studies also suggested that the three compounds were all mixed-types of inhibitors. In addition, compound **6-1** showed better absorption and blood brain barrier permeability. These studies provide better insight into the inhibitory behaviors of DL0410 derivatives, which is beneficial for rational design of AChE and BuChE inhibitors in the future.

## 1. Introduction

Alzheimer’s disease (AD) is the most common neurodegenerative cause of dementia and impacts individual morbidity and mortality significantly, causing a large burden on the health care system [1]. In the case of AD, the proteins implicated include amyloid-beta peptide (Aβ), the microtubule-associated protein tau, the lipotransport protein apolipoprotein E (ApoE) and the presynaptic protein alpha-synuclein [2]. The neurodegenerative cascade is triggered by Aβ deposits in the brain and the hyperphosphorylated tau that aggregates to form the paired helical filaments found in neurofibrillary tangles [3]. Other characteristic pathological features include the loss of neurons and white matter, cerebral amyloid angiopathy, activation of inflammatory processes and the innate immune response, oxidative damage [4,5], mitochondrial dysfunction [6], and low levels of acetylcholine (ACh), which plays critical roles in the peripheral and central nervous systems [7].

The observation that cholinergic markers and cholinergic cells are reduced in the brains of patients with AD led to the so-called cholinergic hypothesis, i.e., the theory that central cholinergic deficiency causes symptoms in AD [8]. Acetylcholinesterase (AChE) inhibition has been the most successful approach to date [9], with four AChE inhibitors and one AChE inhibitor-containing combination having been developed and successfully brought to market. Like AChE, BuChE is a key enzyme involved in the transmission of nerve signals in the brain. In the normal brain, AChE plays an important role in regulating brain levels of ACh, while BuChE accounts slightly for cholinesterase activity. However, in the AD brain, BuChE activity increases while AChE activity remains constant or decreases [10].

At present, four cholinesterase inhibitors (tacrine, donepezil, rivastigmine, and galantamine) were approved for the treatment of AD [11]. There are two substrate-binding sites of the binding pocket of AChE [12], including the catalytic anionic site (CAS), near the bottom of the active site gorge, and the peripheral anionic site (PAS), near its entrance. The active cavity of AChE is located on the upper surface of the enzyme by the composition of a constantly inwardly recessed channel [13,14]. ACh binds to the PAS by electrostatic attraction, and then slides along the narrow channel of the active cavity to reach the catalytic active center of the CAS. Therefore, the PAS site contributes to improving the catalytic efficiency of Ach [15]. Since AChE and BuChE share almost 65% homologic amino acid sequences, BuChE also possesses CAS and PAS [16].

In our previous study, a series of symmetrical molecules containing biphenyl/bibenzyl scaffolds were designed, synthesized, and evaluated for inhibitory activities towards both AChE and BuChE [17]. Among them, DL0410 showed the strongest AChE and BuChE inhibitory activities. The IC_50_ values were 0.096 μM and 1.25 μM, respectively, which was comparable to that of the donepezil, as well as rivastigmine [18]. The patent number of DL0410 was ZL 2007 1 0107604.6. Our previous studies affirmed the efficacy and safety of DL0410 [18,19,20,21]. It was observable that DL0410 had the ability to enhance deficits of memory in scopolamine-induced amnesia in mice by increasing the ACh level in a dose-dependent manner [20]. In addition, DL0410 had an effect on the reduction of Aβ plaques. In APPsw-SY5Y cells, DL0410 regulated the serine-threonine protein kinase (Akt)/Jun N-terminal kinase (JNK) signaling pathway and exerted neuronal protection. In an Aβ-treated mice model, DL0410 reduced Aβ deposits in cerebral cortices, and upregulated neurotrophic brain-derived neurotrophic factor (BDNF)/cAMP-responsive element binding protein (CREB) pathway [18]. DL0410 also improved learning and memory dysfunction in APP/PS1 transgenic mice through cholinesterase inhibition, Aβ plaques inhibition, as well as enhancement of synapse loss [19]. Furthermore, the synthesis route of DL0410 was simple, low-cost, and easy to control. As a result, DL0410 could be regarded as a candidate drug for AD treatment [22].

In the current study, in order to develop more potential drugs against AD, a series of novel AChE/BuChE inhibitors were synthesized based on the skeleton of DL0410 and evaluated for AChE and BuChE inhibitory activities. Based on the inhibition activities, pharmacophore, and three-dimensional quantitative structure activity relationship (3D-QSAR) models were developed for DL0410 derivatives to analyze the relationship between structures and activities. For compounds with high inhibitory activities, kinetic studies and molecular docking were performed to analyze their binding modes and mechanism of inhibition. Intestinal absorption and blood brain barrier (BBB) permeability of strong inhibitory derivatives were also predicted for their druggability, which is very important in drug candidate selection process. These studies provide useful information for rational design of novel AChE/BuChE inhibitors as anti-AD agents in the future.

## 2. Results and Discussion

### 2.1. Inhibiting Activity of DL0410 Derivatives on AChE and BuChE

DL0410 and its 50 DL0410 derivatives were evaluated regarding inhibitory activity of AChE and BuChE by in-house *in vitro* assays. As showed in Table 1, the IC_50_ values of AChE and BuChE had a wide range with 0.03–75.54 μM and 0.06–200 μM ( five of IC_50_ values of BuChE were more than 200 μM), respectively. The IC_50_ values of DL0410 were 0.096 μM and 1.25 μM for AChE and BuChE, respectively. There are two derivatives (compounds **6-1** and **7-6**) with higher AChE inhibition activities than DL0410. The AChE IC_50_ values of compounds **6-1** and **7-6** were 0.06 μM and 0.03 μM, respectively. Their BuChE inhibitory activities of the two compounds also exceeded that of DL0410 with IC_50_ values of 0.61 μM and 0.44 μM, respectively. In addition to the two compounds, the BuChE inhibition activity of compounds **1-8**, **1-9**, **1-15**, **7-3**, **7-5**, and **7-6** was less than 1 μM, and stronger than that of DL0410. From these data, it could be concluded that side chain substituted or biphenyl modified had a great impact on AChE and BuChE inhibitory activities among these derivatives.

### 2.2. Common Feature Pharmacophore Models

**Figure 1:**
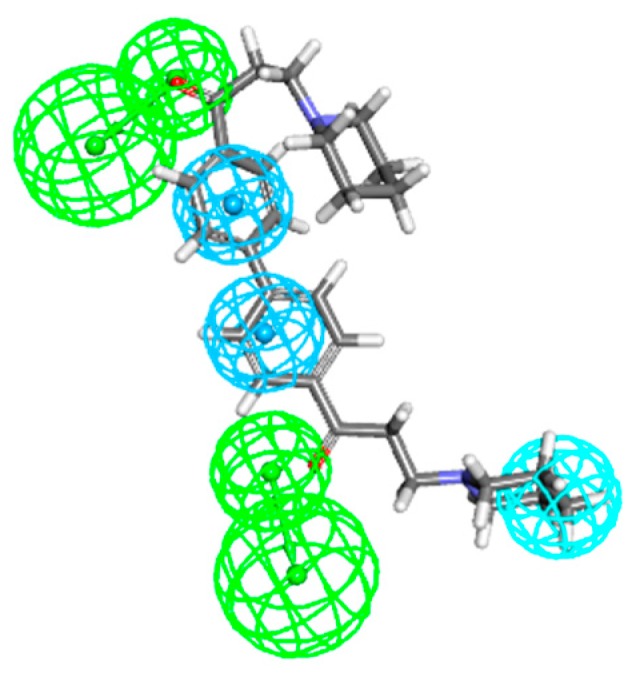
DL0410 showed best alignment with Hypo4 pharmacophore model. Hypo4 consisted of two hydrogen bond acceptors, two ring aromatic features, and one hydrophobic feature.

To have an insight into the relationship of anti-AChE and BuChE activities and structural modifications of these derivatives, we constructed 3D-QSAR models by Discovery Studio 2016 (Accelrys Software, Inc., San Diego, CA, USA). Before building 3D-QSAR models, a common feature pharmacophore model was built first for mole0cular alignment by analyzing the shared spatial arrangement of multiple chemical features between seven different types of active compounds in the training set. The top ten common feature hypotheses (Hypo1–Hypo10) were generated. Then, the ability of these pharmacophores for discriminating active compounds from inactive compounds was evaluated by external data sets. As a result, the best pharmacophore hypothesis (Hypo4) consisted of two hydrogen bond acceptors, two ring aromatic features, and one hydrophobic feature was validated with a good agreement between critical chemical features of these active compounds and ROC (receiver operating characteristic curve) of Hypo4 was 0.925 (Figure 1).

### 2.3. 3D-QSAR Models

**Figure 2:**
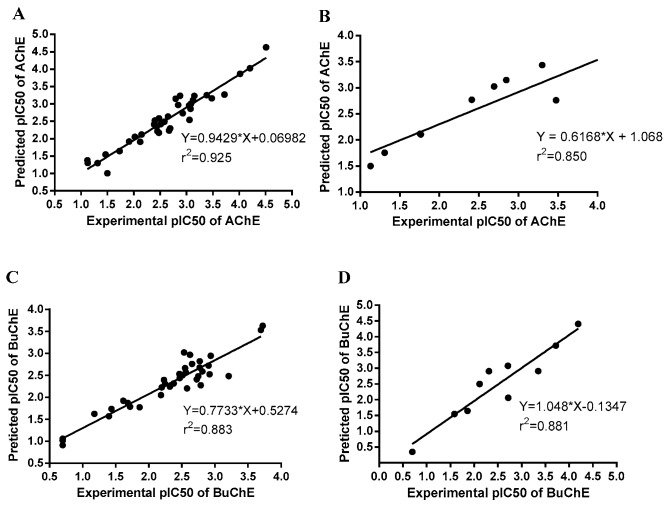
Plot of the experimental versus predicted AChE and BuChE inhibitory activities of the training set and test set. The correlation coefficient R^2^ of AChE and BuChE 3D-QSAR models between determined and predicted activities of training set were 0.925 and 0.883 (**A**,**C**). The R^2^ of AChE and BuChE test set were found to be 0.850 and 0.881 (**B**,**D**), respectively.

The QSAR models were established based on AChE and BuChE inhibitory activities of DL0410 and its 45 derivatives (excluding five compounds with IC_50_ > 200 μM). Totally, DL0410 and its 35 derivatives were used for training set and 10 derivatives for testing set. The IC_50_ values (mol/L) were converted to pIC_50_ values using the formula pIC_50_ = −log IC_50_. Considering the importance of good alignment for 3D-QSAR model, we applied Hypo4 to explore each molecule with best mapping before alignment conformation. The correlation coefficient R^2^ of AChE and BuChE 3D-QSAR models between determined and predicted activity of the training set were 0.925 and 0.883. In order to evaluate the possibility of correlation in the above 3D-QSAR model, the compounds in the test set were used for validation. The R^2^ of test set was found to be 0.850 and 0.881, respectively, which confirmed that the AChE and BuChE QSAR models constructed by us were receivable. The good agreement between the predicted pIC_50_ value and experimental pIC_50_ value for both test sets and training sets are shown in Figure 2.

**Figure 3:**
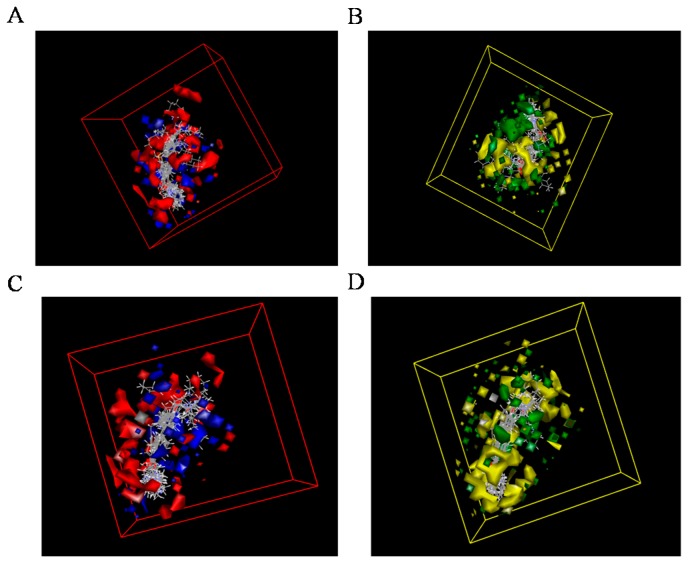
3D-QSAR model coefficients on electrostatic potential and Van der Waals (VDW) grids. Figure **A** and **B** described the coefficients on electrostatic potential grids. Blue represents positive coefficients; red represents negative coefficients. (**A**: AChE, **B**: BuChE). Figure **C** and **D** presented the coefficients on VDW grids. Green represents positive coefficients; yellow represents negative coefficients. (**C**: AChE, **D**: BuChE).

Through the 3D-QSAR model, the relationship between steric or electrostatic molecular fields and activity can be described for guiding the further drug design. A molecule with negative electrostatic potential in the blue area and positive in the red area has a higher activity [23]. Similarly, the 3D view of model coefficients of van der Waals (VDW) interactions is also shown with two colors [24]. Green suggests positive coefficients and yellow indicates negative coefficients. A novel molecule with strong VDW attraction in the green area and weak VDW attraction in the yellow area has a more powerful activity. According to the maps (Figure 3), it suggested that the high negatively-charged substituent group of piperidine and bulky R1/R2 would show lower inhibitory activities towards AChE and BuChE. In addition, 3D-QSAR model of BuChE show more sensitive to electrostatic potential and VDW, validating that nitrogen, oxygen, or bulky substituent group of piperidine were not beneficial for inhibitory activity. For the biphenyl scaffold, a high negatively-charged substituent group and minimized the distance of two benzene rings would help obtain high activity, validating that compounds **6-1** and **7-6** could be more effective.

### 2.4. Kinetics Study and Molecular Docking

**Figure 4:**
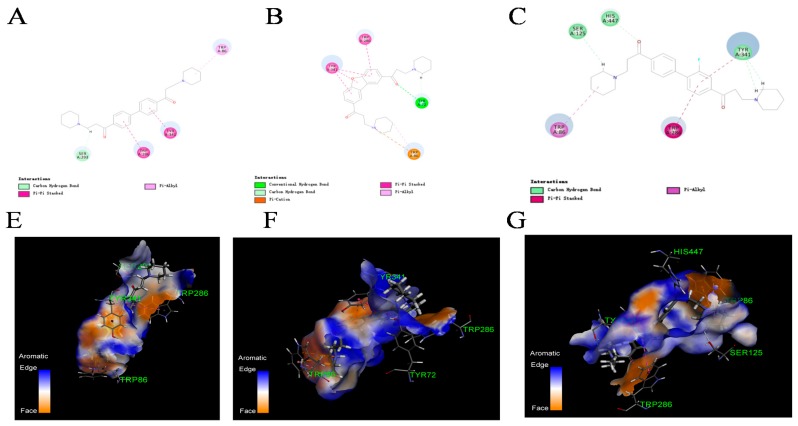
Visualization of the non-bond interactions between the protein and ligands. Figure **A** and **D** showed the interaction between AChE and DL0410. Biphenyl and piperidine rings of DL0410 interacted with TRP286, TYR341 and TRP86 via π-π stacking or π-alkyl interaction. Compound **6-1** showed a strong interaction with AChE (Figure **B** and **E**). Except for TRP286, TYR341 and TRP86, compound **6-1** could form hydrogen bonds with TRP72 via carbonyl group. Compound **7-6** also had powerful interactions with AChE, mainly through π-π stacking, π-alkyl and hydrogen bond interaction (Figure **C** and **F**). TRP286, TYR341 and TRP86 were the key amino acid residues for AChE interacting with the three compounds.

**Figure 5:**
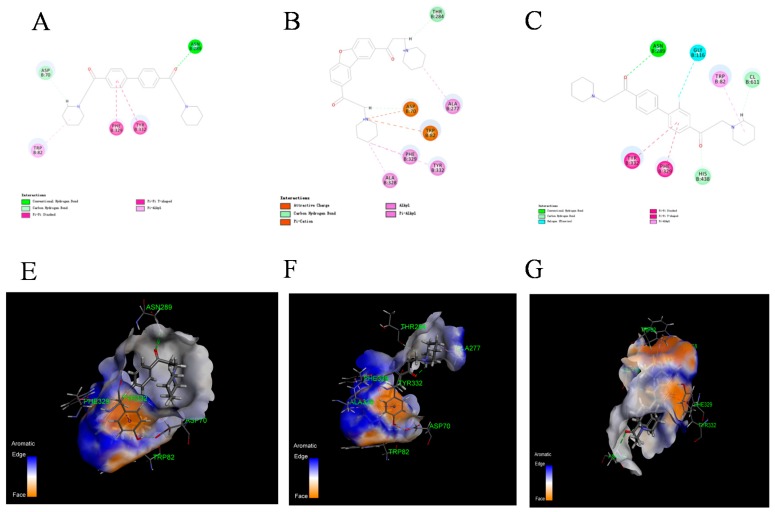
Visualization of the non-bond interactions between the protein and ligands. The figures of **A** and **D** showed the interaction between BuChE and DL0410. Biphenyl and piperidine rings of DL0410 interacted TRP332, PHE329 and TRP82 via π-π stacking or π-alkyl interaction. The carbonyl group could form hydrogen bonds via interacting ASN289. Figure **B** and **E** suggested compound **6-1** had the strongest interaction with BuChE. The piperidine of compound **6-1** had a stronger electrostatic attraction with APS70 and TRP82, and π-Alkyl interaction with ALA328, PHE329, and TYR332. From Figure **C** and **F**, we observed that compound **7-6** also had stronger interaction with BuChE than that of DL0410. There existed more hydrogen bonds for compound **7-6**, and a highly electronegative fluorine atom caused its strong interaction with GLY116.

**Figure 6:**
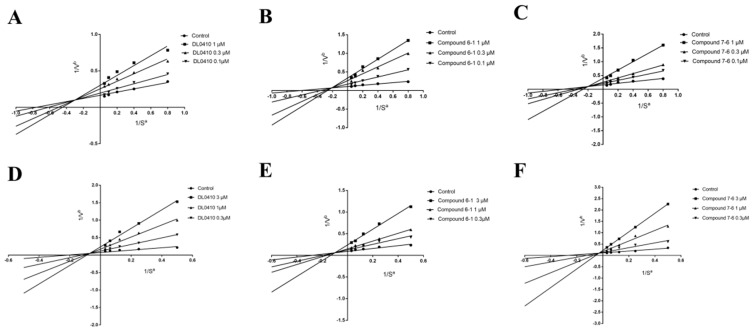
The Lineweaver-Burk plots of DL0410, compound **6-1** and compound **7-6** on AChE inhibitory activity (**A**–**C**). The Lineweaver-Burk plots of DL0410, compound **6-1** and compound **7-6** on BuChE inhibitory activity (**D**–**F**). Kinetic analysis of AChE and BuChE was performed by using three effective concentrations of DL0410, compound **6-1** and compound **7-6** (0.1, 0.3, 1 μM for AChE, and 0.3, 1, 3 μM for BuChE) with the incubation of five different substrate concentrations (1.25, 2.5, 5, 10, 20 mM for AChE, and 2, 4, 8, 16, 32 mM for BuChE). The intersections of fitting lines with different concentrations of the three compounds were neither on the X axis or Y axis of Lineweaver-Burk plots of both AChE and BuChE, suggesting that DL0410, compounds **6-1** and **7-6** were mixed-type of inhibitors. (*n* = 3; a = mM^−1^; b = min/ΔA).

**Figure 7:**
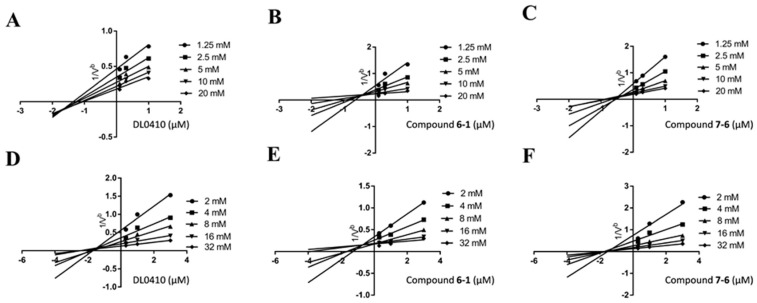
*Ki* values were determined by Dixon plots. Dixon plots of DL0410, compound **6-1** and compound **7-6** on AChE inhibitory activity were showed as Figure **A**–**C**, respectively. The AChE *Ki* values of them were, separately, 1.65 ± 0.14 μM, 0.42 ± 0.04 μM and 0.55 ± 0.2 μM. Figure **D**–**F** presented the Dixon plots of DL0410, compound **6-1** and compound **7-6** on BuChE inhibitory activity, respectively. The BuChE *Ki* values of them were, separately, 1.81 ± 0.08 μM, 0.74 ± 0.16 μM and 1.63 ± 0.18 μM. (*n* = 3; b = min/ΔA).

To have an insight into how structure modification affected the inhibitory activities, molecular docking and kinetics studies were performed for DL0410, compounds **6-1** and **7-6**. After the docking parameters were set, the crystal poses of donepezil and *N*-((1-benzylpiperidin-3-yl)methyl)-*N*-(2-methoxyethyl) naphthalene-2-sulfonamide were first re-docked into the binding site pocket of AChE and BuChE, respectively. Donepezil is a mixed-type inhibitor of AChE used in the palliative treatment of AD [25]. *N*-((1-benzylpiperidin-3-yl)methyl)-*N*-(2-methoxyethyl) naphthalene-2-sulfonamide is a potent, selective, and reversible human BuChE inhibitor, which can improve memory, cognitive functions, and learning abilities [26]. The root mean square deviation (RMSD) values between the docking and initial poses were 0.872 and 1.253, respectively, which implied that the docking models built by CDOCKER could reproduce the crystal binding model and were suitable for the AChE and BuChE system. DL0410 and two highly inhibitory compounds (compounds **6-1** and **7-6**) were docked to the active pockets of AChE and BuChE. For AChE, these three compounds could dock to TRP86 of CAS via π-Alkyl interaction or electrostatic attraction, near the bottom of the active site gorge, and TRP286 of PAS via π-π stacking interaction, near its entrance (Figure 4). The value of –CDOCKER INTERACTION ENERGY was followed by compound **6-1** > compound **7-6** > DL0410. The dibenzofuran of compound **6-1** formed stronger π-π stacking interaction with TYR341 and TRP286, and the carbonyl formed hydrogen bond with TYR72. In addition, hydrogen bond interaction of compound **7-6** made it high inhibition for AChE. The three compounds had a similar interaction with BuChE, and all of them can dock to TYR332 of CAS via π-π stacking interaction, and TRP82 of PAS via π-Alkyl interaction or electrostatic attraction. Compound **6-1** showed the strongest interaction with BuChE, because compound **6-1** changed its extension direction, causing its piperidine to have a stronger electrostatic attraction with APS70 and TRP82, and π-Alkyl interaction with ALA328, PHE329, and TYR332 (Figure 5). There existed more hydrogen bonds for compound **7-6**, and a highly electronegative fluorine atom caused its strong interaction with GLY116. Therefore, the fluorine atom substitution and the structure of dibenzofuran made compounds **6-1** and **7-6** different from DL0410. Compound **7-6** formed more hydrogen bonds with AChE and BuChE. The structure of dibenzofuran caused the extension direction of compound **6-1** to change, which was more conducive to forming electrostatic attractive interactions. *In silico* results were consistent with the kinetics studies of AChE and BuChE. Various concentrations of DL0410, and compounds **6-1** and **7-6** were assayed in order to determine the mechanism and the inhibition constant (*K_i_*) towards AChE and BuChE with a series of substrate concentrations. The inhibition kinetic data were analyzed by Lineweaver-Burk and Dixon plots. The Lineweaver-Burk plot provides a useful means of distinguishing among competitive inhibition, non-competitive inhibition, and mixed inhibition [27], whereas the merit of the Dixon plot is that one can expediently determine *K_i_* from the kinetic data [28]. According to the Lineweaver-Burk plots, we found that the intersections of fitting lines with different concentrations of the three compounds were neither on the X axis or Y axis (Figure 6). This suggested that DL0410, and compounds **6-1** and **7-6** were mixed-type of inhibitors, with AChE *K_i_* values of 1.65 ± 0.14 μM, 0.42 ± 0.04 μM, and 0.55 ± 0.2 μM, respectively (Figure 7). The BuChE *K_i_* values of them were 1.81 ± 0.08 μM, 0.74 ± 0.16 μM, and 1.63 ± 0.18 μM, respectively (Figure 7). The results of the kinetics studies also validated that the three compounds were, indeed, capable of binding to CAS and PAS of AChE/BuChE. The PAS site could improve their catalytic efficiency, which led to high inhibitory effects.

### 2.5. The Evaluation of Absorption and BBB Permeability

According to the prediction results of the PreADMET [29], admetSAR [30], and ADMET descriptor modules in Discovery Studio 2016, we found that the absorption of DL0410, and compounds **6-1** and **7-6** showed no significance by comparing the parameter of Caco-2 permeability and ADMET_AlogP98, and all of them showed moderate absorption levels. However, the parameters of MDCK permeability and ADME_BBB suggested that compound **6-1** crossed the BBB easier than DL0410 and compound **7-6**, and the BBB permeability of compound **7-6** was the most unfavorable (Table 2). Therefore, compound **6-1** had more favorable properties for our further study to verify its efficacy and safety for AD treatment.

## 3. Methods

### 3.1. In Vitro AChE Inhibitory Assay

The AChE inhibitory activity of DL0410 derivatives was assessed by determining the hydrolysis product of Acetylthiocholine iodide (ASCh) using the Ellman’s method [31]. 5,5′-Dithiobis (2-nitrobenzoic acid) (DTNB), and the substrate ASCh were purchased from Sigma Aldrich [20]. AChE was derived from the SD rat brains by homogenizing the brain with 20-fold saline solution, and then centrifuged at 800× *g* for 20 min at 4 °C. To acquire the inhibition of AChE activity, five serial dilutions of samples were measured. A Spectra Max M5 microplate reader (Molecular Devices, Sunnyvale, CA, USA) was utilized for the assay for AChE reaction system with 96-well plates in 0.05 M phosphate-buffered solution (PBS). The reaction system includes 10 μL sample, 20 μL AChE solution, 60 μL 3.75 mM ASCh, and 80 μL 0.25 mg/mL DTNB. It took 60 min at 37 °C for incubation, and the absorbance intensity of AChE reaction system was quantified at 412 nm [19].

### 3.2. In Vitro BuChE Inhibitory Assay

BuChE was obtained from human plasma (purchased from Beijing Red Cross Blood Center China) [19]. Before utilization, human plasma was diluted 200-fold with 0.05 M PBS. Five serial dilutions of samples are determined to obtain BuChE inhibitory activity. The BuChE reaction system is similar to the assay above, including 10 μL samples, 40 μL diluted plasma sample, 70 μL 7.5 mM S-butyrylthiocholine (BuSCh), and 80 μL 0.25 mg/mL DTNB. After incubation at 37 °C for 60 min, the absorbance intensity of the system was measured at wavelength 412 nm.

### 3.3. AChE and BuChE Kinetic Studies

It is important to determinate the type of inhibition in an effort to understand the mechanism of inhibition and the binding sites of the inhibitor sites [32]. Kinetic analysis of AChE and BuChE was performed by using three effective concentrations of DL0410, compound **6-1** and compound **7-6** (0.1, 0.3, 1 μM for AChE, and 0.3, 1, 3 μM for BuChE) with the incubation of five different substrate concentrations (1.25, 2.5, 5, 10, and 20 mM for AChE, and 2, 4, 8, 12, and 24 mM for BuChE). The absorbance intensity was determined at 412 nm every 30 s for 5 min. The effects of the derivatives on AChE and BuChE inhibitory activity were analyzed by a Lineweaver-Burk double reciprocal plot.

### 3.4. Common Feature Pharmacophore Models

The HipHop algorithm in the Discovery Studio 2016 (DS, www.accelrys.com Accelrys, San Diego, CA, USA) [33] was applied to generate common feature pharmacophore models using a training set of 10 AChE and BuChE inhibitors which had a diversity of structure. The features considered in the pharmacophore model generation were H-bond donor (HBD), H-bond acceptor (HBA), hydrophobic (HYA) features, ring aromatic (RA) feature, and ionizable positive (IP). For each compound, possible diverse sets of conformations were generated over a 10 kcal/mol range using the BEST flexible conformation generation option with the maximum conformation of 200. The top-ranking pharmacophores were further validated by testing set to identify the hypothetical orientation of the active compounds and the common binding features interacting with the target.

### 3.5. 3D-QSAR Models

To obtain the structure-activity relationships on the DL0410 derivatives and to identify the more powerful and dual AChE/BuChE inhibitors, 3D-QSAR model was performed by the built-in QSAR software of DS 2016 [34]. Firstly, the corresponding pIC_50_ values were transformed from the acquired IC_50_ (μM) values of AChE/BuChE inhibition. Then, under a CHARMm force field, all the structures were minimized with a root mean squared (RMS) difference of energy gradient reached 0.1 kcal/mol Å. Through the Diverse Molecules method in DS 2016, the compounds were divided into a training set and a testing set. To measure electrostatic and steric effects, two probe types were designed to compute the energy grids which were considered as descriptors for partial least-squares (PLS) model [35]. The population size, maximum generations, and grid spacing were set to be 100, 5000, and 1 Å in acetyl- and butyrylcholinesterase models in the QSAR protocol. Cross-validation was conducted for measuring the predictive performance of a statistical model [36].

### 3.6. Molecular Docking

To identify possible binding mode of DL0410 derivatives, molecular docking of the most potent inhibitors into the active site cavity of AChE and BuChE (PDB code and 4EY7 and 5DYW, respectively) was performed employing CDOCKER protocol of DS 2016. For each inhibitor, conformation with the highest –CDOCKER INTERACTION ENERGY was extracted for further analysis. The original ligands of AChE and BuChE were taken to define the binding site for docking studies. The crystal pose of donepezil in 4EY7 was first re-docked into the binding site pocket of AChE and similarly, *N*-((1-benzylpiperidin-3-yl) methyl)-*N*-(2-methoxyethyl) naphthalene-2-sulfonamide was re-docking into the pocket of BuChE. Then, the root-mean-square deviation (RMSD) values between the docking and initial poses were computed, which implied that the docking model built by CDOCKER could reproduce the crystal binding model.

### 3.7. Absorption, Distribution, Metabolism, and Excretion (ADME)

ADME (absorption, distribution, metabolism, and excretion) properties were very important for evaluating the ability of the drug to reach pharmacologically active concentration at the therapeutic targets. Thus, ADME evaluation can be useful combined to *in silico* models [37]. Drug bioavailability and BBB permeability were key aspects for AD treatment. Therefore, the intestinal absorption and BBB permeability of DL0410 and its derivatives were further predicted. We combined different ADME prediction tools to improve the reliability of the results, including PreADMET server [29], admetSAR server [30], and the ADME descriptors module in DS 2016.

## 4. Conclusions

In this study, a series of novel AChE/BuChE inhibitors were evaluated for their biological activities. Among them, compounds **6-1** and **7-6** exhibited the most potent AChE and BuChE inhibitory activities, exceeding that of DL0410. Pharmacophore and 3D-QSAR models were built with a good correlation coefficient, which contributed to predicting the inhibitory activities for DL0410 analogues quantitatively and uncovering the relationship between steric or electrostatic molecular fields and activity. A docking simulation was performed to determine the potential binding modes and found that the three compounds could interact with both CAS and PAS of AChE/BuChE. Kinetic studies also validated that DL0410, compounds **6-1** and **7-6** were all mixed-types of inhibitors. Compound **6-1** showed better absorption and BBB permeability than DL0410, which could be a potential candidate for AD treatment. Therefore, this study will guide the design of potent AChE/BuChE inhibitors to further strengthen already available drug batch against AD.

## Figures and Tables

**Table 1:** The chemical structures and biological activities of DL0410 and its 50 derivatives as AChE and BuChE inhibitors.

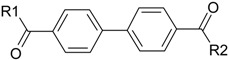				
**Compound No.**	**R1**	**R2**	**IC_50_(μM)**	
			**AChE**	**BuChE**
**1-1**	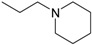	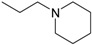	0.096	1.25
**1-2**	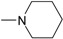	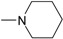	0.84	2.22
**1-3**	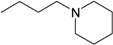	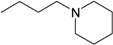	1.32	5.9
**1-4**	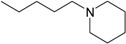	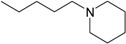	0.82	2.65
**1-5**	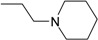	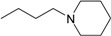	0.41	1.88
**1-6**	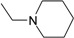	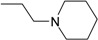	3.32	4.87
**1-7**	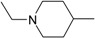	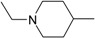	49.05	36.58
**1-8**	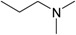	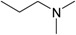	0.75	0.19
**1-9**	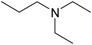	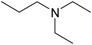	1.18	0.74
**1-10**	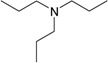	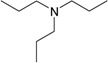	0.88	2.83
**1-11**	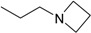	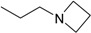	0.71	2.39
**1-12**	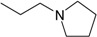	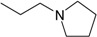	0.33	1.16
**1-13**	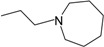	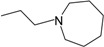	1.43	1.94
**1-14**	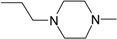	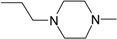	1.61	13.77
**1-15**	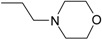	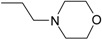	0.97	0.72
**1-16**	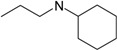	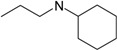	2.15	1.91
**1-17**	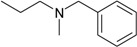	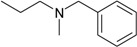	3.33	6.56
**1-18**	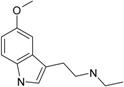	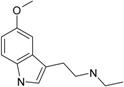	9.52	>200
**1-19**	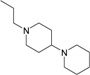	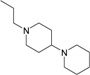	0.25	>200
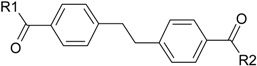				
**Compound No.**	**R1**	**R2**	**IC_50_(μM)**	
			**AChE**	**BuChE**
**2-1**	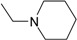	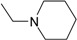	75.54	20.72
**2-2**	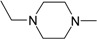	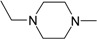	74.13	66.51
**2-3**	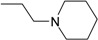	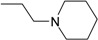	0.50	3.41
**2-4**	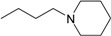	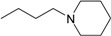	3.67	19.18
**2-5**	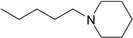	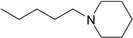	0.19	1.64
**2-6**	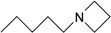	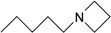	12.24	>200
**2-7**	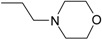	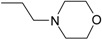	3.53	6.38
**2-8**	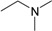	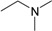	62.27	9.11
**2-9**	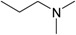	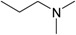	2.62	1.21
**2-10**	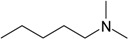	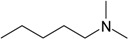	3.96	2.77
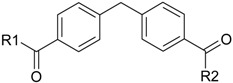				
**Compound No.**	**R1**	**R2**	**IC_50_(μM)**	
			**AChE**	**BuChE**
**3-1**	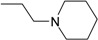	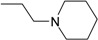	4.11	4.2
**3-2**	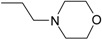	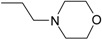	7.09	7.65
**3-3**	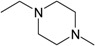	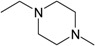	48.53	40.00
**3-4**	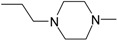	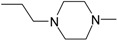	7.55	24.35
**3-5**	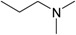	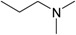	3.32	1.72
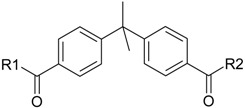				
**Compound No.**	**R1**	**R2**	**IC_50_(μM)**	
			**AChE**	**BuChE**
**4-1**	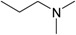	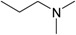	4.89	6.00
**4-2**	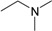	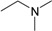	54.44	5.95
**4-3**	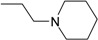	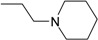	3.07	3.09
**4-4**	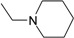	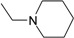	17.40	2.93
**4-5**	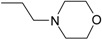	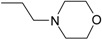	15.67	16.32
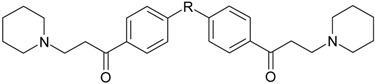				
**Compound No.**	**R**		**IC_50_(μM)**	
			**AChE**	**BuChE**
**5-1**	O		0.89	5.68
**5-2**	S		3.89	3.44
**5-3**	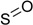		14.08	>200
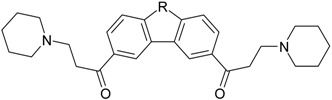				
**Compound No.**	**R**		**IC_50_(μM)**	
			**AChE**	**BuChE**
**6-1**	O		0.06	0.61
**6-2**	S		2.03	1.71
**Compound No.**	**Structure**		**IC_50_(μM)**	
			**AChE**	**BuChE**
**7-1**	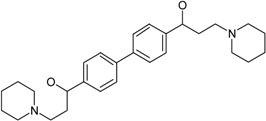		31.74	13.78
**7-2**	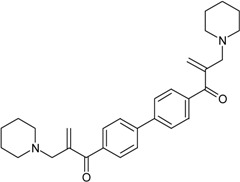		17.13	1.55
**7-3**	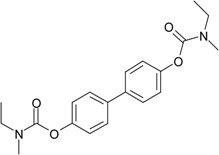		34.09	0.06
**7-4**	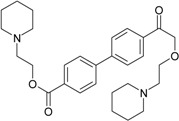		18.53	>200
**7-5**	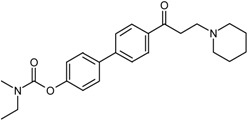		3.35	0.20
**7-6**	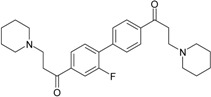		0.03	0.44
**7-7**	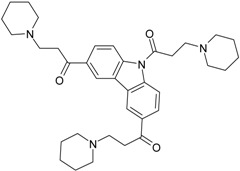		1.42	4.79

**Table 2:** The ADME properties predicted by the PreADMET and ADMET modules in Discovery Studio (DS) 2016 and admetSAR.

ADMET Prediction Tool	ID	DL0410	Compound 6-1	Compound 7-6
PreADMET	Caco-2 permeability (nm/s)	55.75	49.58	57.90
	MDCK permeability (nm/s)	0.18	4.88	0.91
ADMET module in DS	ADMET_AlogP98	5.17	5.38	2.03
	ADMET_BBB	0.79	0.85	−0.37
admetSAR	Caco-2 permeability (LogPapp, cm/s)	1.11	1.10	0.68
